# Automated Cough Assessment on a Mobile Platform

**DOI:** 10.1155/2014/951621

**Published:** 2014-08-10

**Authors:** Mark Sterling, Hyekyun Rhee, Mark Bocko

**Affiliations:** ^1^Department of Electrical and Computer Engineering, University of Rochester, Rochester, NY 14627, USA; ^2^School of Nursing, University of Rochester, Rochester, NY 14627, USA

## Abstract

The development of an Automated System for Asthma Monitoring (ADAM) is described. This consists of a consumer electronics mobile platform running a custom application. The application acquires an audio signal from an external user-worn microphone connected to the device analog-to-digital converter (microphone input). This signal is processed to determine the presence or absence of cough sounds. Symptom tallies and raw audio waveforms are recorded and made easily accessible for later review by a healthcare provider. The symptom detection algorithm is based upon standard speech recognition and machine learning paradigms and consists of an audio feature extraction step followed by a Hidden Markov Model based Viterbi decoder that has been trained on a large database of audio examples from a variety of subjects. Multiple Hidden Markov Model topologies and orders are studied. Performance of the recognizer is presented in terms of the sensitivity and the rate of false alarm as determined in a cross-validation test.

## 1. Introduction

The use of sound information is a growing area of application of signal processing techniques in healthcare and biomedicine. In particular, multiple groups have reported on the development of systems that analyze audio recordings of patients and identify bouts of coughing [1–5] or other sounds indicative of health concerns [6–9]. In addition to their value as a monitoring or screening tool, the automated nature of these systems potentially enables new modes of disease management, especially in conjunction with advances in mobile technology. Moreover, the type of approach presented here may be especially suited to improving outcomes and addressing chronic disease in populations that have been previously underserved [10].

This paper studies cough as a symptom of asthma in adolescents. For a variety of reasons, including social and developmental pressures, adolescents with an asthma diagnosis typically have an inaccurate understanding of their disease condition primarily due to poor symptom perception or downplaying symptoms [11, 12]. These issues can undermine effective symptom monitoring and medication adherence which can ultimately lead to poor condition management and high healthcare utilization. To address this problem a device is proposed that continuously monitors asthma symptoms, particularly coughing which is the most common symptom in pediatric asthma patients [13, 14]. The identified symptoms can be stored and later retrieved by a patient or healthcare provider as an objective reference indicating the levels of asthma control.

Mobile technology has advanced sufficiently such that the hardware requirements of a personal asthma monitoring device are already met in existing consumer electronics platforms. The problem may, therefore, be approached within the context of mobile application development. Owing to its broad install base and mature software development kit (SDK) Apple's iOS devices were the target platform (the 4G iPod touch specifically).

Like other audio systems for cough monitoring reported in the literature the present approach is based upon proven technology and methods in the fields of speech recognition, keyword and key audio effect detection [15–17], machine learning, and content-based audio analysis [18–23]. The symptom recognition algorithm consists of a frame-based feature extractor followed by temporal analysis with a Hidden Markov Model (HMM) based Viterbi decoder. This is a commonly employed framework for speech recognition tasks [24]. Mel-frequency cepstral coefficients (MFCC) plus a log-energy measure were employed as the audio features while both left-to-right and* ergodic*/connected HMM topologies were investigated. The MFCC/HMM recognition framework has also been reported for cough monitoring by, for example, Matos et al. [3, 25]. Shin et al. [2] use a similar method however; in their case, they use an original variant of cepstral analysis dubbed energy cepstral coefficients (ECC) and have inserted an artificial neural network discriminator prior to the HMM decoder.

## 2. Methods

The cough detection algorithm presented here processed the audio data in two steps. In the initial preprocessing step the incoming stream of digital audio samples was rearranged into fixed-length frames from which a set of audio features (feature vector) were computed. The sequence of feature vectors was then passed to a HMM Viterbi decoder (using a token passing implementation as described in [24]) using HMMs trained on the collected database of symptom and background sounds. As discussed in detail in the following sections the HMM was a composite consisting of separate models for* coughing*,* background*, and* silence* connected together with a simple parallel grammar.

### 2.1. Data Collection

Prior to development of the Automated Device for Asthma Monitoring (ADAM) application, a large amount of audio data was collected in trial recordings intended to closely approximate the circumstances in which the device would be expected to function. Subjects were recruited from the emergency department and pediatric outpatient clinics. Participants with asthma were 13–17 years old, had active asthma symptoms within the previous 24 hours, and used English as a primary language. Those with other health conditions producing respiratory symptoms (e.g., cold, cardiac disease, or cystic fibrosis) were excluded. Twenty-nine adolescents participated in the audio data collection trials; details of the sample characteristics are provided elsewhere [26].

Participants with asthma were 13–17 years old, had active asthma symptoms within the previous 24 hours, and used English as a primary language. Those with other health conditions producing respiratory symptoms (e.g., cold, cardiac disease, or cystic fibrosis) were excluded.

The participants were instructed to continuously record the sounds of their respiration for 24 hours* in vivo* wearing a personal digital recorder (Olympus WS-331 M) and lapel microphone while they went about their daily routines. The external microphone was attached to the shirt collar to optimally capture the respiratory sounds of the subject. The recorder was carried in a pocket or in a carrying case provided by the study. Subjects paused the recording when privacy was desired. Before going to bed the subjects were instructed to place the recorder and microphone near the head of the bed to capture any symptoms that occurred during the night.

**Figure 1:**
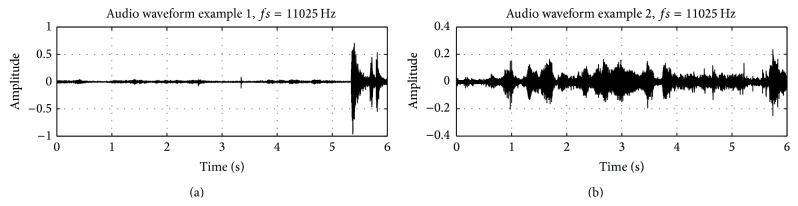
Representative audio waveforms from database. The top trace shows a long segment of silence followed by a cough. The bottom trace shows a segment of background noise. These are 16-bit PCM.wav files at a sampling rate of 11025 Hz.

Each individual provided at minimum 10 hours of data. The database thus represented many hours of recorded audio. For the purposes of developing the signal processing component of the ADAM device the large dataset was split into a series of files of more manageable size. Representative selections of the audio containing cough sounds, silence, and background noise across all of the participants were aggregated into individual files. The classification and validation of symptomatic sounds were carried out by the principal investigator and three other clinicians trained in pediatric medicine and pulmonology. Coughing sounds that were used to train the recognition algorithm were cropped to only include the sound itself. As a consequence the corresponding audio files had variable length (i.e., the duration of the cough). All other audio files (including the training data for the silence and background HMMs) had a fixed duration of 6 seconds. Two example waveforms from the audio database are shown (Figure 1). The files were standard mono 16-bit pulse code modulation (PCM) audio format with a sample rate of 11025 Hz.

To keep training times manageable (especially for the HMMs with a large number of mixture components and states) representative portions were selected from the audio database totaling approximately 2 hours for the training of* background* and* silence* HMMs. The cough model was trained on 110 examples of isolated coughs. Test datasets were also created to evaluate the performance of the classifiers. The performance metrics that were of most practical interest, given the task, were the false positive rates *R*_FP_ and true positive rates *R*_TP_ (sensitivity). To estimate *R*_FP_, approximately 6 hours of representative, cough-free, background noise was compiled. To estimate *R*_TP_ the recognition algorithm was tested on 30 examples of cough that were withheld during training.

### 2.2. Preprocessing

The preprocessing step consisted of dividing the time-domain digital audio data into frames and then computing a sequence of per-frame descriptive feature vectors. The MFCCs are among the commonest features used in speech and audio applications. These are implemented natively by the open source Hidden Markov Toolkit (HTK) which was used to train the symptom recognition algorithm.

All signal processing operations were implemented at a sampling rate of 11025 Hz. Frame size was chosen to be 256 samples overlapping by one-half of a frame. The overall frame rate was thus 0.0116 seconds. To compute the MFCCs features a 256-point Hanning window multiplied the given frame of audio samples and the DFT of the result was computed. The results were stored in an array *A*[*k*].

**Figure 2:**
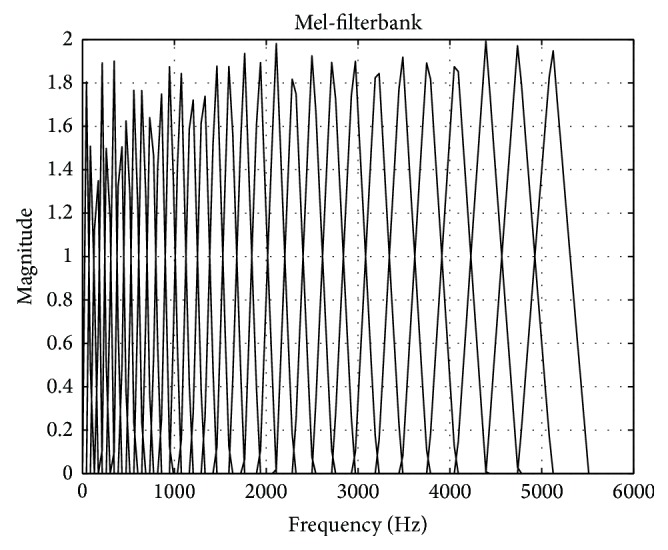
32-point Mel-filterbank used in the feature extraction step.

A 32-point mel-filterbank (Figure 2) was applied to the magnitude of the spectrum stored in *A*[*k*]. In practice, setting array sizes to an appropriate power of 2 greatly simplifies programming when discrete Fourier transforms are needed. The logs of the 32 filterbank magnitudes were computed and another DFT was performed. Importantly, the indices of *A*[*k*] were reordered to introduce the symmetries needed to compute a discrete cosine transform (DCT) with a DFT. The result of the second DFT was stored in an array *C*[*k*] and the DCT coefficients were found via the following formula:

(1) $$\begin{array}{c} \text{MFCC}\left[k\right]=\frac{R\left\{q\left[k\right]\right\}R\left\{C\left[k\right]\right\}-I\left\{q\left[k\right]\right\}I\left\{C\left[k\right]\right\}}{2}. \end{array}$$

Here, *q*[*k*] is a fixed array arising from the definition of the discrete cosine transform. For *k* ∈ (1,31)  *q*[*k*] is equal to a complex exponential with an additional factor of $1/\sqrt{2}$ when *k* = 0:

(2) $$\begin{array}{c} q\left[k\right]=\{\begin{array}{cc} \frac{2}{\sqrt{2N}}e^{-i\pi k/(2N)}, & k\in \left(1,31\right),\\ \frac{2}{2\sqrt{N}}e^{-i\pi k/(2N)}, & k=0. \end{array} \end{array}$$

Twelve MFCCs were used for the audio feature vector. This corresponded to the above formulas for *k* ∈ (1,12). The zeroth cepstral coefficient is representative of the overall energy in the frame. This value was not retained because a different* log-energy* measure was used instead to track sound intensity. This measure was defined as follows [24]:

(3) $$\begin{array}{c} E=\log\overset{N-1}{\underset{n=0}{\sum }}x^{2}\left[n\right]. \end{array}$$

The base of the logarithm is *e*, *N* is the frame size equal to 256, and *x*[*n*] are the values of the time-domain audio samples. Although the computation of the mel-cepstrum is not an invertible operation it is reasonable to assume, based upon the performance of the classifier, that these MFCCs contained enough discriminative acoustic information for the task. As stated above, the zeroth MFCC is an energy measure. In speech recognition, the first MFCC is understood to roughly distinguish between sonorants and obstruents. The higher MFCCs, however, are not known to admit any simple perceptual interpretations [27, 28].

**Figure 3:**
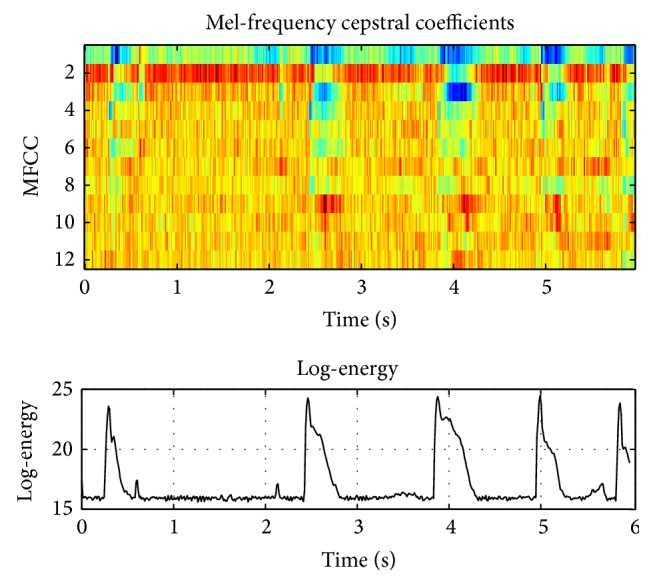
Illustrative example of the audio features (MFCCs and log-energy) seen by the classifier.

The first twelve MFCCs and the log-energy defined one-half of the feature vector for each frame. An example of these features for a given frame is shown (Figure 3). The other half of the feature vector was defined to be the first differences of the MFCCs and log-energy. If *i* is the index of the frames in time then the first difference of any quantity *f* at time *i* is defined as (*f*_*i*+1_ − *f*_*i*−1_)/2. At frame boundaries the difference was computed between neighbors. For simplicity in eventually transferring the recognition algorithm to a mobile application a preemphasis filter was not applied.

### 2.3. Sound Modeling

Hidden Markov Models [23] are often used to describe a sequence of observations as the probabilistic evolution of a finite state machine. Associated with each state is a probability distribution that describes the elements of the observation vector. This is used to calculate a conditional likelihood for a given observation during decoding. The sequential nature of the relevant data or process is captured in the state-to-state transition probabilities.

HMMs are partially specified as directed graphs and two example topologies are shown (Figure 5). The nodes in the graph represent states and the arrows represent allowed transitions between states. The exact topology appropriate for a given problem must usually be found through experimentation. Two topologies were examined: an ergodic or connected topology and a left-to-right topology (5). Following [15], connected models were used for background and silence HMMs while both the connected and left-to-right topologies were tested for identifying coughs (the key effect). Clearly, for a fixed number of states, left-to-right models are much more constrained than connected models as quantified by the total number of possible paths through the model. Comparing the two, the connected models are expected to have a higher *R*_FP_. This basic trend is borne out in experimental results (Table 1).

**Figure 4:**
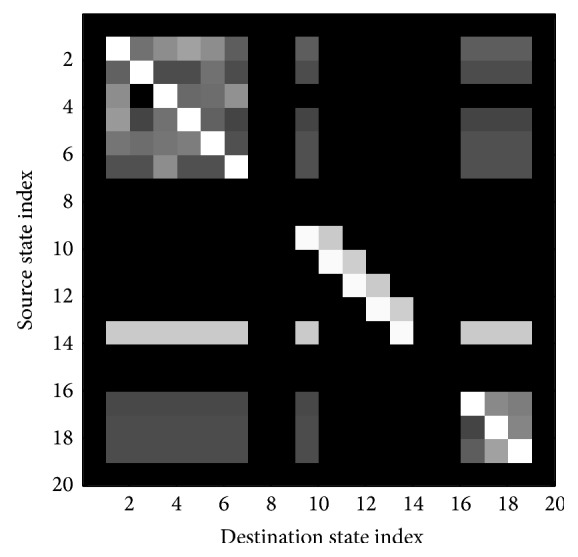
Image of the transition map of the composite HMM. The matrix has a block structure with each block corresponding to one of the sound classes,* background*,* cough*,* silence*. The intensity of each pixel in this image corresponds to the log-probability *p*_*ij*_ for a token to pass from the row to the column. The off-diagonal blocks correspond to transitions between the various sound classes.

**Figure 5:**
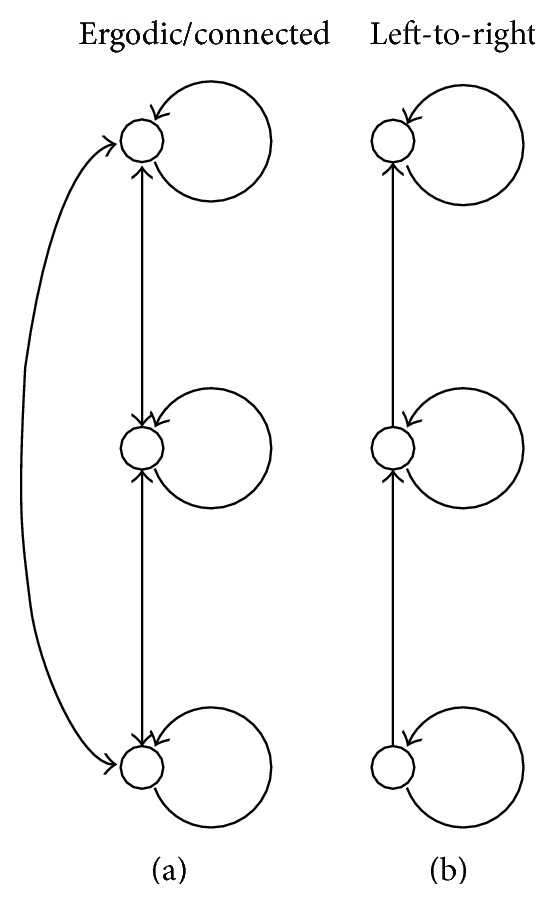
The ergodic/connected HMM topology (a) and the left-to-right HMM topology (b). The connected topology allows a transition from one state to any other state. The left-to-right topology only allows a transition to the same state or to the next state up.

**Figure 6:**
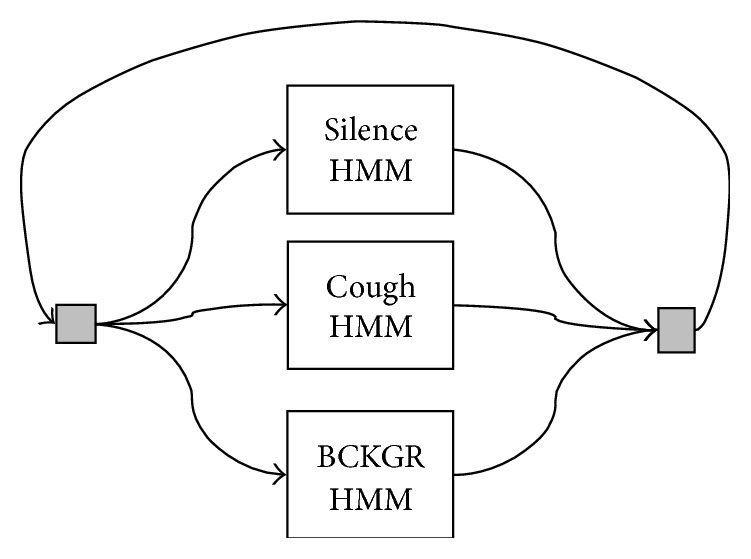
Grammar network used for constructing a composite HMM from* silence*,* cough*, and* background* HMMs. This parallel structure is the most simple and freest way to combine the individual HMMs. The gray boxes represent dummy states that route the outputs to the inputs (and, importantly, do not cost a time unit).

The role of context partially determines the grammar for the recognition task (equivalently, context determines how the background, silence, and cough models should fit together in a composite HMM). Generally, for coarse segmentation of the audio stream, contextual information was not expected to greatly improve the performance of the classifier. This contrasts with speech where context can provide a significant assistance for recognition and even help to overcome poor phoneme level transcription (i.e., possible sequences of phonemes are constrained by the vocabulary of the language and, moreover, the sequence of words is constrained by grammatical rules). Therefore, individual HMMs were fitted into a parallel HMM (Figure 6). The gray boxes at the left and right indicate dummy states that route all of the output states to all of the input states so that the system can be run in a continuous fashion. Ultimately, the structure of the composite HMM reflects in the transition matrix *p*_*ij*_ of the model. A graphical representation of this matrix is shown (Figure 4).

In addition to the topology, an HMM is also specified by an output probability distribution in the feature space for each state of the model. Usually, for a given HMM, these probability distributions all have the same functional form. For the recognizer, the output probability distributions were Gaussian mixtures with diagonal covariance matrices. The feature vector is denoted by **f** = [*f*_1_,…, *f*_*M*_]^*T*^. A single multivariate gaussian probability distribution *b* is

(4) $$\begin{array}{c} b_{f,\mu ,\Sigma }\left(f\right)=\frac{1}{\sqrt{{\left(2\pi \right)}^{M}\left|\Sigma \right|}}\exp\left(-\frac{1}{2}{\left(f-\mu \right)}^{T}\Sigma^{-1}\left(f-\mu \right)\right). \end{array}$$

The mixture model is a weighted sum of such terms:

(5) $$\begin{array}{c} B_{f}\left(f\right)=\overset{J}{\underset{i=1}{\sum }}\alpha_{i}b_{f,\mu_{i},\Sigma_{i}}. \end{array}$$

The total number of mixture components is denoted by *L* in this case.

The* silence* model used in this paper was a 3-state connected model with 3 mixtures per state. For* background*, a variety of connected HMMs were tested with the number of states ranging over the values (3,6) and the number of mixture components per state ranging over the values (10,30,60). Likewise, for* cough*, connected and left-to-right topologies were tested with 5 states and the number of mixtures ranging over the values (10,60).

After selecting the orders of the HMMs the models were trained. As stated above, the HMMs reported on in this paper were trained with tools provided in the Hidden Markov Toolkit (HTK) [24]. Specifically, each HMM was initialized using a single pass of* Viterbi* training until convergence followed by a single pass of* Baum-Welch* training until convergence. In the nomenclature of HTK this is accomplished with the command line programs HInit and HRest, respectively (using the “whole-word” strategy).

### 2.4. Recognition by Viterbi Decoding

To recognize a new audio stream Viterbi decoding was applied with the composite HMMs described in previous sections. The Viterbi decoding is implemented with* token passing* [24, 29]. In the recognition context the behavior of Hidden Markov Models is best understood using a lattice where the horizontal axis is the discrete time index and the vertical axis an index of the states. A token can be visualized as an entity which moves through the state lattice and keeps a record of the path and the likelihood of the path it has taken. The likelihood is simply the sum of the appropriate values of the emission probabilities *B*(**f**_*n*_) (where *n* is a timing variable at the frame rate) and the state-to-state transition probabilities *p*_*ij*_.

Viterbi decoding finds the most likely path through the lattice given the data. Using the token idea the decoding algorithm can be stated simply. The following two rules must be adhered to. (*Rule*1) To update a new frame of data, or equivalently to move to the next time step in the lattice, compute all possible transitions for the current set of tokens; (*Rule*2) for each state keep the incoming token that has the highest likelihood. In general, if there are *N* frames of data and *L* states then arrays of size *N* × *L* must be kept in memory. In particular, the likelihoods and the states where the tokens originated are retained in memory. To recover the actual state alignment the algorithm begins at the end of these arrays and works backwards, following the path of the most likely token.

The update rule can also be stated mathematically as a recursion [29]:

(6) $$\begin{array}{c} s_{j}\left[n\right]=\underset{i}{\max}\left(s_{i}\left[n-1\right]+\log p_{ij}\right)+\log B_{j}\left(f_{n}\right). \end{array}$$

**Figure 7:**
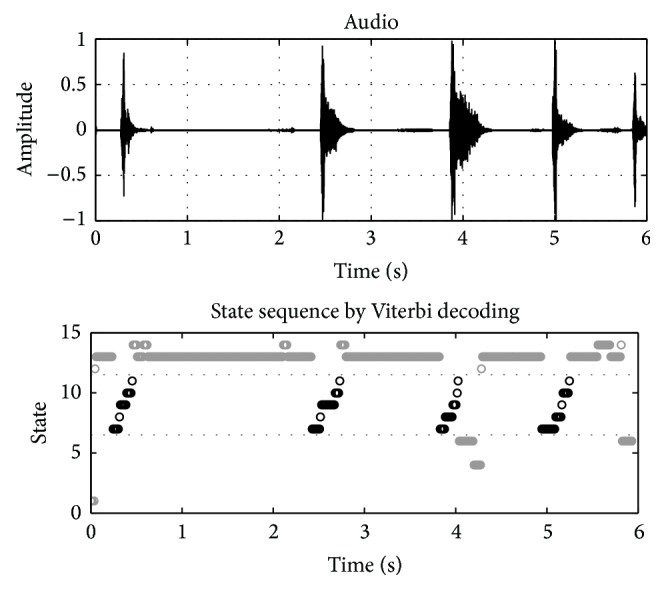
Example Viterbi decoding pass for audio containing coughs. In this case the background model had 6 states with 60 mixtures per state while the cough model had 5 states with 60 mixtures per state with a left-to-right topology. The horizontal axis represents time (at the frame rate) while the vertical axis is an integer of the state index. Gray marks indicate states associated with the* silence* and* background* models. Black marks indicate states associated with the cough model.

An example of a decoding pass for some audio data containing coughs is presented (Figure 7) (note that for continuity this is the same audio data shown above (Figure 3)). A time-domain waveform of the audio being decoded and the resultant state sequence are shown (Figure 7). The horizontal axis represents time (at the frame rate) while the vertical axis is an integer of the state index. The lighter gray marks indicate states associated with the* silence* and* background* models. The black marks indicate states associated with the* cough* model (in this case, a left-to-right topology). After the Viterbi decoder, the rule for deciding whether a frame of audio data contains coughing is simply whether the state sequence ever passed through the cough model. This is equivalent to observing when the maximum likelihood path passed through the middle branch of the composite HMM (Figure 6). Note that when the* cough* model uses a left-to-right topology it forces any detected cough event to have visited each state of the cough HMM in a linear manner which is observed in the state sequence (Figure 7). As discussed briefly above, this constraint partially explains why the rates of false positive are generally better for the left-to-right versus connected* cough* models.

### 2.5. Implementation Notes

As stated in the introduction, the ultimate goal of this project was to create a symptom recognition application for asthmatics that could run, continuously, on a mobile device. Consequently, Apple developer credentials were obtained and the recognition algorithm described above was implemented in the Xcode software development environment and iOS software development kit. Standard Core Audio APIs were used for interfacing to the audio hardware of the mobile platform (4G iPod touches). All of the required signal processing was facilitated by the Fast Fourier Transform routines provided in the Accelerate framework [30]. Moreover, custom cabling assemblies were constructed that allowed Olympus ME-15 tie-clip microphones to interface with the iPod.

Numerical underflow (or overflow) is a potential pitfall of working with HMMs and the Viterbi algorithm. Equations (4) and (5) show that the probabilities involved can quickly accrue large exponents especially for a greater number of features and especially in conjunction with the Viterbi algorithm. Therefore, computations are carried out, where possible, with log-likelihoods. By taking care of computing the probability for each mixture component it was found that 64-bit floating point arithmetic could be used without underflowing. The exact algorithm for this computation is provided below. Again, the feature vector was 26-dimensional consisting of twelve MFCCs, the log-energy measures, and the first differences of these quantities. Here, **f**, ***μ***, **v**, and *α* are, respectively, the feature vector, the mean vector for the given component, a vector of the values along the main diagonal of Σ^−1^, and the mixture weight.

The likelihood for the entire state is found by running Algorithm 1 for each associated mixture component and then taking the natural log.

**Figure 8:**
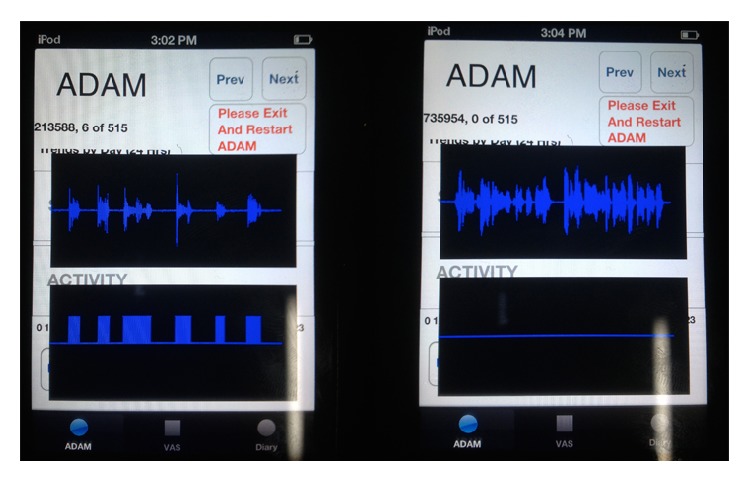
Screenshots of the ADAM application showing live capture and processing of audio data. The top traces are time-domain audio waveforms and the bottom traces indicate the presence of coughs. The left screenshot shows a sequence of coughs being recognized while the right screenshot shows some example speech being discounted. This is a demonstration mode used for debugging that is normally inaccessible to patients; hence the interface is somewhat rough. The total length of audio represented is 6 seconds in each screenshot. The middle number at the upper-left is the actual cough count which is determined by finding the instances where the last* cough* state is followed by the first state of any model in the grammar.

Two example instances of the mobile ADAM application running in a demonstration mode that was used for debugging are shown (Figure 8). This mode was inaccessible to patients. The data presented in the screenshots is similar to the Viterbi decoding examples shown earlier (Figure 7) with the exception that the audio is being obtained and processed live on a mobile device. The top trace is the time-domain audio waveform and the bottom trace is a consolidation of the corresponding maximum likelihood state sequence found by token passing. Let *a*_seq_[*m*] for 0 < *m* < 514 be maximum likelihood state sequence (for the chosen frame rate, 6 second blocks of audio result in 515 feature vectors). From *a*_seq_ another sequence is created that is defined as

(7) $$\begin{array}{c} a_{\text{cough}}\left[m\right]=\{\begin{array}{cc} 1, & a_{\text{seq}}\left[m\right]\in \text{coughstate},\\ 0, & a_{\text{seq}}\left[m\right]\in \text{otherwise}. \end{array} \end{array}$$

Thus *a*_cough_[*m*] indicates where coughs occur in the audio according to the recognition algorithm. The left screenshot shows the application identifying a sequence of coughs from the database while the right screenshot shows the device discounting some nonsymptom speech (Figure 8). These tests were conducted in an anechoic environment with the test sounds played through high-quality active speakers. This is not to claim that such tests recreate the conditions of a patient wearing the device in a nonlaboratory setting. Rather, the tests were meant to verify that the recognition framework and application could return positive results in circumstances where any concerns over the reproducibility of test signals were minimized. It should be stated here that the performance measures given in the next section were determined by cross-validation trials with the test database. The logical next step in evaluating the device is to trial it with a population of patients. Such a test will be a subject of future work. Such a trial will not only measure the accuracy of the recognition algorithm but will also measure the scope of the test database (i.e., how well the algorithm generalizes).

## 3. Results

Performance results for different model orders are presented in Table 1. The columns in Table 1 are from left to right as follows: the number of states in the* background* model, the number of mixtures per state for the* background* model, the number of states in the* cough* model, the number of mixtures per state for the* cough* model, the topology of the* cough* model, the rate of false positive per 1 hour, and the sensitivity. The* silence* HMM for these trials was constant. It is also noted that the number of states listed in Table 1 follows an HTK convention of including the dummy states. Therefore, the number of states that are actually active during decoding is the given number minus two.

The classification algorithm was a two-step process. Firstly, 6 seconds of PCM audio was collected and split into frames with 256 points and half a frame overlap. Feature vectors for each frame were computed and the audio was segmented in time by a Hidden Markov Model based Viterbi decoder. The entire 6 seconds of audio was classified as either containing a cough or not containing a cough based upon the criterion explained above (Figure 7).

In the classification task the data is skewed since it was expected that* cough* would occur relatively infrequently when compared to the frequency of other background noises. Here, overall error rate would not be the most illuminating performance measure. Instead, a pair of metrics was used: the rate of false positive (or false alarms) per 1 hour FP/1 h and* sensitivity* or true positive rate. The rate of false positive is found by counting the number of false positives and dividing by the total length of time that was analyzed (in units of hours). This can be estimated robustly since it is not a particular challenge to collect large volumes of audio data that are free of coughs (i.e., any hit is guaranteed to be a false alarm, and these are easy to count). Let TP, TN, FP, and FN be, respectively, the total number of true positives, true negatives, false positives, and false negatives returned by a classifier. The sensitivity is defined as

(8) $$\begin{array}{c} \text{Sensitivity}=\frac{\text{TP}}{\text{TP}+\text{FN}}. \end{array}$$

Sensitivity measures how often the classifier misses symptoms. It was estimated using a holdout trial. The composite HMM was tested with 30 examples of cough that were withheld during training.

## 4. Discussion

The results presented in the previous section show that certain model topologies and complexities provide a reliable detection and segmentation of cough and background sounds. Clearly, the prototype used for the* cough* HMM has a powerful effect upon both performance measures. As expected, the connected* cough* models result in a higher false alarm rate (keeping the other variables fixed). Also, it is noted that the* cough* model requires a larger number of mixture components per state to achieve a decent rate of false positive. In trying to select among these alternative algorithms the ultimate application must be kept in mind. The goal is to provide data to the asthma patients that can give them a clearer picture of their disease state. A significant rate of false alarm will negatively impact the user's trust in the feedback provided by the device. Thus, aiming for low FP/1 h a large* background* HMM and a left-to-right* cough* HMM were used.

Previous studies have proposed systems for automated ambulatory monitoring of cough. Matos et al. describe the Leicester Cough Monitor (LCM) [3, 25]. Like the system presented here, the LCM uses a recognition system consisting of preprocessing to calculate the cepstral features followed by HMM analysis. A very high sensitivity (85.7%) and low false positive (0.8 per hour) rate were achieved using this approach. However, in the LCM the audio analysis is performed offline and the ambulatory component of the study consisted strictly of an audio recorder. They report that a 24-hour audio recording takes approximately 1 hour to process. This differs critically from the present study where all of the processing is performed on the mobile device and the results are provided as feedback in real time. The inherent time and computational restrictions in the present study prevented the use of HMMs of the same complexity (in terms of the number of hidden states) as the LCM. Like ADAM and the LCM, Shin et al. [2] report on a cough detection system that uses HMM analysis of audio features. Instead of using cepstral features directly Shin et al. processed cepstral features with an artificial neural network before passing them to the HMM Viterbi decoder. While their system successfully discriminated cough from environmental noise it was only validated using synthetic examples at most 10 minutes in length so it is not directly comparable to the* in vivo* situation that is of concern here. Also, the present study is the first to examine the feasibility of ambulatory cough detection on a consumer mobile device in an adolescent asthma population.

Effective asthma management relies upon the accurate monitoring of symptoms by patients. However, current monitoring techniques have had limited success with adolescent groups. Around their peers, adolescents with asthma may feel embarrassed about their condition and may also be reluctant to take medication when needed. The ADAM device is intended to improve self-management for these patients. ADAM provides reliable and objective symptom monitoring throughout the day that can be accessed privately through an attractive mobile interface. Also, since ADAM is deployed on an already popular consumer platform, the device can be used inconspicuously and the self-management benefits it provides will not be hampered by social pressures. Acceptance of ADAM by adolescents has been established previously with qualitative surveys [26]. Overall, it is expected that adolescents who have access to a technology such as ADAM will demonstrate greater adherence to treatment regimens, better awareness of symptom severity and, in the long term, require fewer emergency room visits.

Important limitations of this study are also noted. Although cough is the most common symptom found in pediatric patients with uncontrolled asthma [13, 14] there are other respiratory symptoms such as wheeze and the initial intention was to design a device to monitor both coughing and wheezing. However, wheeze was encountered infrequently during the data collection phase and the examples of wheeze that were recorded showed significant audio variations between each other. This frustrated attempts to design a reliable algorithm for identifying wheeze and distinguishing it from background noise. In principal, the methods described that were used for detecting cough could be applied to wheeze but it would require a more representative audio database than was available in the present study.

There are also practical limitations to consider. Firstly, because ADAM continuously records and processes sounds it is more resource intensive than typical current generation mobile applications. This impacts battery life negatively and may interfere with other applications that may be running on the device concurrently. Future iterations of the application with better optimized code and power management will be needed to address these concerns.

Although mobile technology is becoming increasingly ubiquitous ADAM is still not appropriate for all situations. The device generally needs to be carried on the person and the lapel microphone must be positioned appropriately. Certain activities, such as sports, may require the patient to set the device aside or the patient may be dressed in a manner that makes the ADAM device awkward to carry (i.e., clothing without a convenient pocket). Moreover, in certain social settings, an adolescent may not want to be seen wearing a microphone. These practical limitations are expected however; as long as patients make reasonable effort to use ADAM whenever possible the device will provide benefit in terms of asthma self-management.

## 5. Conclusion

Using a large audio database collected in a controlled study an algorithmic approach to the automated assessment of coughing in adolescent asthmatics was explored. This work represents the technical development of a device (ADAM) capable of providing objective round-the-clock feedback to adolescents with an asthma diagnosis. The eventual goal is to leverage the semantic information that can be gleaned from an audio stream to benefit the patient. In conjunction with the user interface capabilities and also the ubiquity of modern mobile phones this work enables new standards in 24-hour asthma symptom monitoring.

## Figures and Tables

**Algorithm 1:**
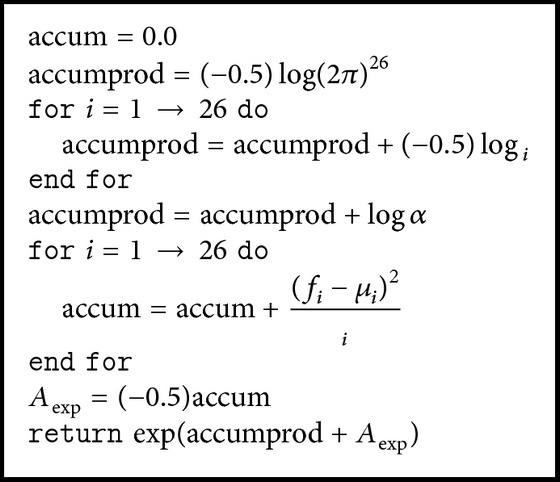


**Table 1:** Rate of false positive (per 1 hour) and sensitivity (true positive rate for hold-out trial).

S_BGR_	Mix_BGR_	S_COH_	Mix_COH_	Top_COH_	FP/1 h	*R*_TP_
5	10	7	60	LTR	3	0.63
5	10	7	60	CON	32	0.80
5	30	7	60	LTR	2	0.70
5	30	7	60	CON	26	0.87
8	10	7	60	LTR	1	0.63
8	10	7	60	CON	20	0.80
8	30	7	60	LTR	1	0.60
8	30	7	60	CON	10	0.77
8	60	7	60	LTR	1	0.57
8	60	7	60	CON	11	0.83
5	30	7	10	LTR	413	1.00
8	60	7	10	LTR	106	0.97
8	60	7	10	CON	609	1.00
